# Unpredictable Chronic Mild Stress Suppresses the Incorporation of New Neurons at the Caudal Pole of the Chicken Hippocampal Formation

**DOI:** 10.1038/s41598-019-43584-x

**Published:** 2019-05-09

**Authors:** F. Gualtieri, E. A. Armstrong, G. K. Longmoor, R. B. D’Eath, V. Sandilands, T. Boswell, T. V. Smulders

**Affiliations:** 1 Centre for Behaviour & Evolution, Newcastle University, Newcastle upon Tyne, UK; 2 Institute of Neuroscience, Newcastle University, Newcastle upon Tyne, UK; 3 School of Natural and Environmental Sciences, Newcastle University, Newcastle upon Tyne, UK; 4 Animal and Veterinary Science Research Group, SRUC, Edinburgh, UK

**Keywords:** Evolution, Adult neurogenesis, Stress and resilience

## Abstract

In the mammalian brain, adult hippocampal neurogenesis (AHN) is suppressed by chronic stress, primarily at the ventral pole of the hippocampus. Based upon anatomy, we hypothesise that the caudal pole of the avian Hippocampal Formation (HF) presents a homologous subregion. We thus investigated whether AHN is preferentially suppressed in the caudal chicken HF by unpredictable chronic mild stress (UCMS). Adult hens were kept in control conditions or exposed to UCMS for 8 weeks. Hens experiencing UCMS had significantly fewer doublecortin-positive multipolar neurons (*p* < 0.001) and beaded axons (*p* = 0.021) at the caudal pole of the HF than controls. UCMS birds also had smaller spleens and lower baseline plasma corticosterone levels compared to controls. There were no differences in AHN at the rostral pole, nor were there differences in expression of genetic mediators of the HPA stress response in the pituitary or adrenal glands. Duration of tonic immobility and heterophil/lymphocyte (H/L) ratios were also not responsive to our UCMS treatment. These results support the hypothesised homology of the caudal pole of the avian HF to the ventral pole of the rodent hippocampus. Furthermore, quantifying neurogenesis in the caudal HF post-mortem may provide an objective, integrative measure of welfare in poultry, which may be more sensitive than current welfare measures.

## Introduction

In mammals, the hippocampus is a well-defined and highly plastic forebrain structure^1^ which is sensitive to various conditions and experiences. Much recent evidence supports the existence of a functional gradient across the longitudinal axis of the rodent hippocampus^2^, perpendicular to major subfields including the dentate gyrus and Ammon’s Horn^3^. Whilst the dorsal region appears critically involved in cognitive processing domains such as spatial memory and navigation^4–6^, the ventral region mediates emotional responses through modulating activity of the hypothalamic-pituitary-adrenal (HPA) axis^7,8^. Consistent with these putative specializations, activation of granule cells specifically in the dorsal or ventral dentate gyrus differentially suppresses either contextual learning or innate anxiety, respectively^9^. Gene expression profiles^10^ and neural connectivity^11^ also differ between the sub-regions. Anatomical connectivity supports the existence of a functionally homologous longitudinal axis in the hippocampus of humans and other primates^2^, indicating that this organisation is conserved across the mammalian lineage.

The dentate gyrus is one of few discrete areas in the mammalian brain where adult neurogenesis, the production and integration of new neurons across the lifespan, occurs^12^. Adult hippocampal neurogenesis (AHN) differs across the dorso-ventral axis in terms of baseline levels of cell proliferation and maturation^13^. The number of new-born cells produced through AHN is also responsive to environmental factors: it is decreased by chronic negative stress^14^, but increased by experiences generally associated with positive mood, such as exercise^15^, environmental enrichment^16^ and antidepressant treatment^17^. Collectively, these findings suggest that levels of AHN may be modulated by long-term affective state in a valence-specific manner: upregulation with positive states, and downregulation with negative states. In addition, sensitivity of AHN to these stimuli varies along the dorso-ventral axis. Several experiments have found that reductions in AHN which result from chronic stress in rodent models of depression occur preferentially at the ventral pole of the hippocampus^17–21^. Correspondingly, many (though not all) antidepressants restore levels of AHN in the ventral rodent hippocampus^18,19,22–24^. This mechanism of action has a causal role in re-establishing normal behaviour, as anhedonia persists following monoaminergic antidepressant treatment in mice subject to hippocampal irradiation, or with a transgenic mutation disrupting the neurogenic pharmacological response^25,26^. Similarly, unlike in wild-type mice, recovery of stress-induced behavioural submissive symptoms fails to occur upon transfer to an enriched environment in transgenic mice with AHN selectively ablated^27^. As demonstrated by a recent study, adult-born neurons act to inhibit the activity of mature granule cells in the ventral dentate gyrus which are preferentially activated under anxiogenic conditions^28^, thus playing a causal role in resilience to chronic stress. Concurrent to this, environmental regulation of AHN entails integration of sequentially occurring positive and negative experiences, which points to potential application of the level of AHN as a marker of cumulative welfare^29^.

Whether the avian Hippocampal Formation (HF; combining hippocampus proper and area parahippocampalis *sensu* Karten & Hodos^30^) is similarly responsive to chronic stress and positive experiences has not yet been widely explored. Developmentally, the avian HF is derived, like the mammalian structure, from the dorsomedial portion of the telencephalon^31^. However, unlike the mammalian hippocampus, it is not covered by more laterally-derived parts of the telencephalon (i.e. cortex), and therefore is located on the surface of the brain. Avian and mammalian hippocampal formations are widely accepted to be homologous, with roles in spatial memory and navigation^32–34^. With regards to emotional modulation, lesion and stimulation work in pigeons has demonstrated that the avian HF, like the mammalian hippocampus, also exerts negative feedback on to the HPA-axis^35^. Overall external connectivity of the avian and mammalian HFs is largely equivalent^36^, but internal cytoarchitectural differences are notable as the avian HF lacks an identifiable dentate gyrus and Ammon’s horn^32^. However, as the functional gradient of the rodent hippocampus lies perpendicular to these subfields, exploration of a similar axis within the avian HF does not depend on characterisation of their homologues. Anatomically, both the dorsal region of the rodent hippocampus and the rostral region of the avian HF connect to the septum^36^. Thus, we hypothesise homology between these two areas, and hence also between the caudal pole of the avian HF and the stress-responsive ventral hippocampal sub-region in rodents^37^. Consistent with this notion, electrical stimulation of the pigeon HF has been demonstrated to suppress plasma corticosterone (CORT) titres more dramatically when performed at caudal sites than at more rostral points^35^. This suggests that the avian caudal pole may indeed be the primary origin of hippocampal negative feedback inhibition to the HPA-axis, as characterises the ventral rodent hippocampus.

Though adult neurogenesis is fairly widespread in the avian telencephalon^38^, there is evidence to suggest that its modulation by external factors within the HF resembles that in rodents. In terms of cognitive enrichment, spatial memory challenge afforded by opportunities to cache and retrieve food upregulates both proliferation and the number of surviving new neurons in the avian HF^39^. Furthermore, a group of studies in chickadees (*Poecile atricapillus* & *P. gambeli*) demonstrate that AHN is reduced in wild-caught birds placed in captivity^39,40^ (though see^41^) but not suppressed in birds hand-reared in captivity^42^, when compared to free-living conspecifics. This suggests that down-regulated AHN in the former birds is a result of stress provoked by the transition to captive conditions, rather than a consequence of the relatively impoverished captive environment^43^. This latter effect may instead be manifest in HF volume, which is lower in both wild-caught and hand-reared captive birds compared to free-living birds^42^. Additionally, in diurnal Indian house crows (*Corvus splendens*), AHN is suppressed as a consequence of 2 weeks’ constant light exposure^44^, likely constituting a chronic stressor. Finally, AHN levels are lower in commercial broilers subject to chronic food restriction than counterparts fed *ad libitum*^45^. Evidence that the avian HF is sensitive to various sources of stress is therefore accumulating.

Nevertheless, few studies have exposed birds to experimental chronic stress protocols mirroring rodent models, and nobody has yet measured consequent levels of AHN with respect to position across the rostro-caudal axis. Reduction of the number of new-born neurons in response to stress would support homology with mammalian mechanisms which modulate hippocampal plasticity. In addition, observed suppression of AHN which was more pronounced at the caudal HF pole would support the hypothesised homology of this subregion with the rodent ventral region. Measurement of the neurogenic stress response in poultry has potential application to animal welfare in terms of facilitating comparison of the cumulative chronic stress experienced by commercial birds in different housing systems and husbandry conditions.

To quantify the number of surviving new-born cells generated through AHN, we stained sections for doublecortin (DCX), an endogenous protein marker of migratory immature neurons in the mammalian^46^ and avian^47^ brain. We predicted that the number of DCX-positive immature neurons would be decreased in hens exposed to UCMS as compared to control birds, and this effect would be more pronounced at the caudal pole. Data were also collected on commonly-used measures of chronic stress, including baseline plasma CORT titres, heterophil/lymphocyte (H/L) ratios and durations of Tonic Immobility (TI), a freezing response which reflects anxiety in poultry^48^: both to assess the impact of our stress treatment and to determine whether changes were associated with, or independent of, AHN. Finally, we measured expression levels of a number of genes related to HPA-axis function in pituitary and adrenal tissue, predicting alterations comparable to those observed following restraint stress in laying hens^49,50^.

## Methods

### Ethical statement

The experiment was granted ethical approval by the Animal Welfare and Ethical Review Body at Newcastle University and Scotland’s Rural Colleges. All procedures were conducted according to local and UK Home Office regulations (Project Licence PPL 60/4192).

### Animals

Given their widespread use in the poultry industry, Hy-Line brown layer hens were selected for this study. On 24 June 2014, 15-week-old pullets (n = 64) were obtained from a commercial rearing farm and transported to the animal facility. Mean body mass upon arrival was 1278 g (±85 g). Birds were randomly assigned to pens of four, each measuring 1.95 m (L) × 0.89 m (W) × 1.20 m (H), with four pens per climate chamber. The climate chambers were 3.90 m (L) × 2.45 m (W) × 2.17 m (H) and allowed for detailed programming of both light cycles and temperature, which was set to a baseline of 21 °C with 40–60% relative humidity. The initial photoperiod was set at 10 h light per day (light:dark ratio, L:D = 10:14 h) to match the rearing farm light programme, and then was incrementally increased to 14 h light per day (L:D = 14:10 h) by 19 weeks of age, as per industry guidelines, before the experiment commenced. Brightness was set to 20–40 lux at bird height. One plastic nest box (0.30 m (W) × 0.49 m (L) × 0.40 m (H)) with three solid sides, a roof and a solid floor that was lined with litter was provided per pen, along with a perch in the form of a cuboid wooden rail (0.03 m (W) × 0.90 m (L)). Wood shavings were used as litter and were replaced weekly, in addition to being topped up once in between bedding changes. Food (Poultry Breeder (HPB) Pellets from Special Diets Services, UK) and water were provided *ad libitum*. Birds were identified via numbered leg rings. The experimental protocol is summarised in Table 1.

**Table 1:** Experimental timeline with respect to age of the chickens in weeks. UCMS = unpredictable chronic mild stress protocol.

Age (wks)	15	16	17	18	19	20	21	22	23	24	25	26
Tonic immobility	X		X							X		
Blood sampling				X		X					X	
Pre-treatment		xxx	xxx	xxx								
UCMS					xxx	xxx	xxx	xxx	xxx	xxx	xxx	xxx
Tissue Collection												X

During the first week post-arrival, TI times were measured for each bird according to the test procedure described below. This index of inherent anxiety levels^51^ informed allocation of birds to experimental conditions based on matching average TI times, thus controlling for individual differences in stress predisposition. Animals were divided equally between the control (n = 32) and UCMS treatment (n = 32) groups and re-allocated to pens of four birds across four different chambers. Birds were distributed evenly between the pens based on TI times and body mass measured on arrival. All eight pens in two of the chambers were assigned to the UCMS treatment, with the remaining two chambers allocated to the control treatment. All hens from three pens each in the control and UCMS chambers (both one pen in one chamber and two in the other) were assigned to be treated with CORT in the drinking water (20 mg/l). Samples from these animals were treated exactly like the other birds and processed alongside them, but the results are not reported because the CORT treatment had deleterious effects on the physical health of these birds and was therefore terminated after 3 weeks. The results that follow are therefore based on the 40 birds (5 control pens and 5 UCMS pens) that were never treated with CORT. Given that hippocampal tissue was collected for multiple purposes, tissue from 12 control and 12 UCMS birds was allocated for immunohistochemistry.

In order to ascertain pre-treatment measures for the hens once introduced to their experimental housing conditions and social groups, birds were left largely undisturbed for a 3-week period after assignment to their social groups. In these three weeks, TI times were again measured (pre-treatment week 2) and baseline blood samples were taken (pre-treatment week 3).

### Unpredictable chronic mild stress

The 8-week experimental phase began 4 weeks after arrival of birds at the facility (age 19 weeks). Throughout this time, control animals were generally left undisturbed, apart from during weekly pen cleaning, when they were temporarily placed in individual cardboard boxes (0.45 × 0.25 × 0.25 m) for about 30 min. In contrast, the UCMS group were exposed to one stressor per day of the 8-week period (detailed in Table 2), in a sequence which was randomised in terms of both time of day and frequency of stressor recurrence. Additionally, the photoperiod in the UCMS chambers was randomised throughout the 8-week protocol, with the 14 hours of light broken up into multiple shorter intervals which varied daily.

**Table 2:** Stressors employed in randomised daily sequences during the UCMS protocol.

Stressor	Definition
Temperature	Standard temperature of 21 °C was unpredictably set either at 12 °C or at 30 °C for 3 h
Short day	4 h photoperiod (L:D = 4:20 h)
Short night	21 h photoperiod (L:D = 21:3 h)
Chased by humans	An experimenter physically chased animals for 5 min, repeated every 30 min for 4 h in total. Animals were seized and released several times within each 5 min session
Wind	An oscillating fan was placed in the middle of each chamber for one day
Food deprivation	Food, but not water, was removed from each pen overnight (12 h)
Wet litter	Litter was saturated with a water hose without animal removal and left wet overnight
Nest box removal	Nest boxes were removed from the pen for 5 h
Social mixing	Animals from different pens and chambers were mixed randomly in a separate chamber with 4 pens in it for 2 h
Isolation	Animals individually constrained in a transportation box (0.45 × 0.25 × 0.25 m) for 2 h

### Tonic immobility

All birds in our study were subjected to the TI test on arrival, pre-treatment and during the sixth week of the experimental phase, to assess the anxiogenic impact of stress treatment over time. Briefly, TI was induced by an experimenter overturning a chicken onto its back in a restraint box (0.45 × 0.25 × 0.25 m) and restraining it with one hand on the chest and the other covering the eyes, until struggling ceased. Induction was considered successful if the bird remained lying down for at least 10 seconds after the hands were removed. The number of attempts required to induce TI and the duration of the TI state were recorded from the moment the hands were removed until the bird righted itself. In order to minimise disturbance to other birds, the TI test was conducted in a separate room from the climate chambers. Where immobility was not induced after 4 attempts, a time of 0 seconds was recorded. A maximum time of 10 minutes was allowed.

### Blood sampling

Blood samples were collected at three time points during the experiment: (1) before the stress manipulation, (2) two weeks into the stress manipulation and (3) during the penultimate week of stress treatment, for quantification of H/L ratios and baseline CORT. Briefly, in a separate room birds’ masses were recorded and blood was collected from the brachial vein within 2 min of capture. 1.5 ml of blood was drawn from the vein with a 23G syringe needle from each animal. One drop was smeared on to a glass microscope slide for later quantification of heterophils and lymphocytes; the rest was centrifuged (15 min, 2000 rpm) to separate plasma from the blood cells. Samples were stored at −80 °C until use for plasma CORT measurement.

### ELISA on plasma CORT levels

The total amount of CORT in chicken plasma was determined in each experimental group using a Corticosterone ELISA kit (Enzo Life Sciences Cat# ADI-900-097, RRID:AB_2307314) according to the manufacturer’s instructions. Briefly, plasma samples were thawed and a five-point standard curve from the supplied CORT (200,000 pg/ml) was created with the following concentrations: 20000, 4000, 800, 160 and 32 pg/ml. To optimise CORT concentration for our assay we referred to Wada *et al*.^52^, running an optimization protocol in which plasma dilution and steroid displacement buffer (SDB) concentration were optimised using stripped, spiked plasma diluted into a buffer of known CORT concentration (~500 pg/ml). Samples were run against a standard curve at plasma dilutions of 1:10, 1:20, 1:40 and 1:60, each with 0%, 1%, and 2% SDB (% of raw plasma volume). For the ELISA of chicken plasma, a CORT dilution of 1:40 or greater with 1% SDB (per raw plasma volume) was sufficient to eliminate measurable effects of plasma on the assay. Optimizing SDB concentration is critical as higher concentrations appear to degrade antibody activity in the assay, artificially increasing estimated CORT amounts in wells. To avoid variability between plate runs affecting the within-bird comparisons, the three samples taken from the same animal were run simultaneously in the same 96-well plate. Samples from different treatments were equally distributed across 3 plates and measured in singlicate. Each well contained: 10 µl of sample (1:40 dilution), 10 µl of 1:100 Steroid Displacement Reagent (SDR) and 380 µl of ELISA assay buffer. For the first incubation with conjugated CORT and antibody, the plate was shaken for 2 hours (500 rpm at 25 °C). For the second incubation with substrate solution, the plate was incubated at the same temperature for 1 hour, without shaking. After adding stop solution, optical density was read with a Multiskan Ascent plate reader (Thermo Fisher Scientific, UK) at 405 nm, with correction between 570 and 590 nm according to the manufacturer’s instructions. Values for each plate were then analysed with My Assays software (Brighton, UK) using a ready-to-use Excel macro designed specifically for the ELISA assay employed.

### Heterophil and lymphocyte quantification

Increased ratio of heterophil to lymphocyte (H/L) white blood cells is considered a reliable indicator of stress^53^. Smears collected from all birds during blood sampling were sent to the Easter Bush Pathology lab (The Royal [Dick] School of Veterinary Studies - University of Edinburgh) for staining and quantification in order to obtain H/L ratio profiles.

### Tissue collection

Over the final four days of the UCMS protocol, animals were killed by intravenous injection with pentobarbital according to a schedule alternating between treatment groups and pens. Immediately thereafter, the pituitary gland was extracted from the skull, after which the brain was dissected from the skull and transferred to a petri dish filled with ice-cold 0.1 M PBS. The hemispheres were divided along the longitudinal fissure with a scalpel and either the left or right hemisphere of 12 control and 12 UCMS birds was fixed for immunohistochemistry in an alternating manner (see below for details of the fixation protocol). The HF was extracted from the remaining hemisphere and allocated for molecular biology. Both HFs were extracted from the remaining 8 birds from each treatment for further molecular analyses (results reported elsewhere). At the same time, the spleen, liver and right adrenal gland were dissected from the abdomen. All tissues used for RNA extraction were weighed, snap-frozen in dry ice and stored at −80 °C.

### Quantitative real-time PCR

RNA was extracted from adrenal and pituitary samples using TriSure reagent (Bioline, London, UK) and Lysing Matrix D tubes in a FastPrep Instrument (MP Biomedicals, Cambridge, UK). In combination with DNAse treatment using the Ambion DNA-Free kit (Thermo-Fisher, Loughborough, UK), 2 µg RNA was reverse transcribed using the Tetro™ cDNA Synthesis Kit (Bioline, London, UK) for use in quantitative real-time PCR (qPCR).

Gene specific primers were designed using the primer-BLAST tool (National Center for Biotechnology Information) and sequence information is displayed in Table 3. Melanocortin 2 receptor (*MC2R*), Steroidogenic Acute Regulatory Protein (*STAR*), 3 beta-hydroxysteroid dehydrogenase (*HSD3B2*) and proopiomelanocortin (*POMC*) mRNA levels were quantified in tissue from the adrenal gland, whilst mRNA for *POMC*, corticotrophin releasing hormone receptor 1 (*CRHR1*) and vasotocin 4 receptor (*VT4R*) was quantified in the pituitary. The chicken lamin B receptor (*LBR*) gene was used as a housekeeping gene to normalise target gene expression, as employed previously^54^. *LBR* expression did not differ between the two treatments in the current study in either the pituitary (*t*_37_ = 0.703, *p* = 0.486) or the adrenal gland (*t*_37_ = 0.571, *p* = 0.572).

**Table 3:** Gene-specific primers used for qPCR in adrenal and pituitary tissue.

Gene	Accession	Orientation	Primer Sequence (5′ - 3′)	Product Length (bp)
*LBR*	NM_205342	Forward	GGTGTGGGTTCCATTTGTCTACA	80
		Reverse	CTGCAACCGGCCAAGAAA	
*MC2R*	NM_001031515	Forward	ATTGCCTCACTGCCAACCAG	160
		Reverse	AGCAGGCACAGTAAGGGTTG	
*StAR*	NM_204686.2	Forward	AGTGATGGCCCTTATCTCGGT	103
		Reverse	GGTGGCTGCTACAAACACTGC	
*HSD3B2*	NM_205118.1	Forward	GCCAAAGAGGAGCAAACCAG	203
		Reverse	TTCACCTCAGTCTTGCCCTG	
*POMC*	NM_001031098	Forward	ATTTTACGCTTCCATTTCGC	141
		Reverse	AATGGCTCATCACGTACTTGC	
*CRHR1*	NM_204321	Forward	CCCTGCCCCGAGTATTTCTA	141
		Reverse	CTTGCTCCTCTTCTCCTCACTG	
*VT4R*	NM_001110438	Forward	GGTTGCAGTGTTTTCAGAGTCG	137
		Reverse	CAAGATCCGCACCGTCAAG	

Standards were produced by gel purification of PCR products using the MinElute gel extraction kit (Qiagen Ltd, Crawley, UK) and their concentration was measured with a NanoDrop spectrophotometer (Thermo Fisher Scientific, Loughborough, UK). Serial dilutions of standards were produced to create standard curves for absolute qPCR quantification. Real-time PCR reactions were run on a Bio-Rad CFX-Connect real-time PCR machine (Bio-Rad, Watford, UK) using the conditions: 95 °C for 2 min, followed by 40 cycles of 95 °C for 5 s, 60 °C for 10 s, and 72 °C for 15 s. Real-time PCR reactions (20 μl) were run using 5 μl of cDNA template together with 10 μl SYBR green master mix (No-ROX kit, Bioline, London, UK) and gene specific primers (400 nm). No-template controls were also included. Standard curves were run in duplicate and pituitary and adrenal samples were each run in singlicate in single assays on a 96-well plate. A melting curve analysis was performed to confirm specificity of the PCR reaction. Assays were analysed using CFX-Manager software (Bio-Rad, Watford, UK) and target gene expression was expressed as a ratio in relation to *LBR* expression measured in the same samples.

### Immunohistochemistry

Brain hemispheres (n = 24) were immersion fixed for 44–48 h in 4% paraformaldehyde in 0.1 M Phosphate Buffered Saline (PFA - PBS) at 4 °C. They were then cryoprotected in a solution of 30% sucrose in 0.5 M PBS before being embedded in OCT (4583, Electron Microscopy Sciences - USA). Coronal sections (50 μm) were cut on a cryostat (HM 550, Microm – Germany) and stored in cryoprotectant solution (30% glycerol and 30% ethylene glycol in 0.1 M PBS). Serial sections taken at 400 μm intervals were then processed for immunohistochemistry.

Free-floating sections were washed in 0.1 M PBS at room temperature, endogenous peroxidase was inhibited for 30 min in 1% H_2_O_2_ in dH_2_O and tissue was permeabilised for 1 h in 0.1 M PBS containing 0.1% Triton X-100, 1% bovine serum albumin and 2% normal horse serum. Samples were then incubated overnight (16 h) with a primary antibody raised in goat against DCX (Santa Cruz Biotechnology, Cat# sc-8066, RRID: AB_2088494) at 1: 500 dilution. The following day, after washing three times in PBS, sections were incubated for 2 h at room temperature with biotinylated horse anti-goat IgG (Vector Laboratories, Cat# BA-9500, RRID: AB_2336123). Sections were then incubated at room temperature for 1 h with horseradish peroxidase streptavidin (Vector Laboratories, Cat# SA-5004, RRID: AB_2336509) and stained using the avidin–biotin complex indirect technique with diaminobenzidine tablets (D4418 SIGMAFAST™ tablets - Sigma Aldrich) as chromogen^55^. Brain samples were then rinsed in water, mounted on gelatin-subbed slides, dried on a pre-warmed hotplate at 37 °C and coverslipped with mounting medium (03989 Eukitt, Sigma Aldrich) for image analysis.

### Stereological quantification

**Figure 1:**
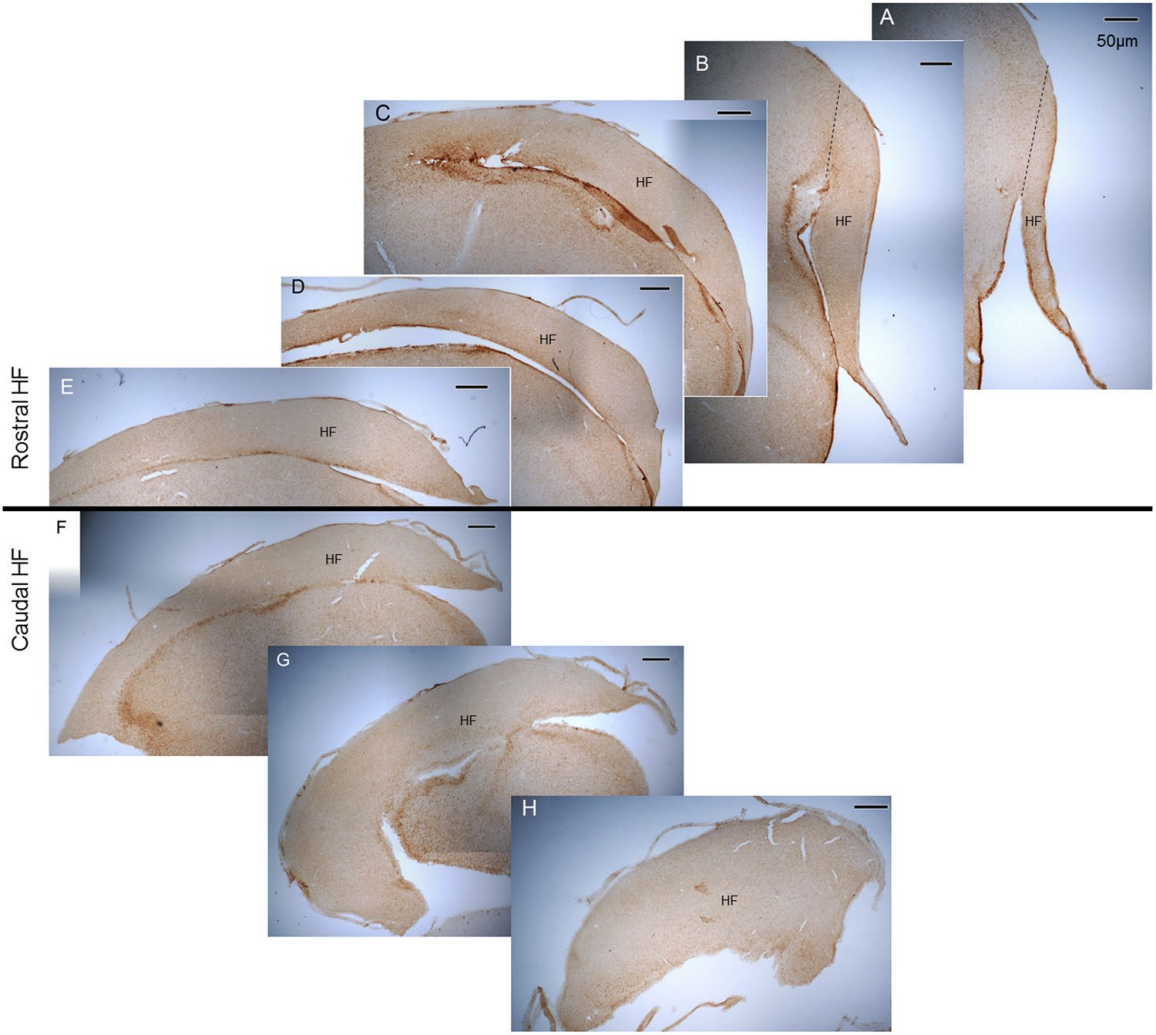
Representative 50 µm thick DCX-stained coronal sections containing rostral (**A–E**) and caudal (**F–H**) hippocampal formation (HF). Rostral sections are 1600 µm apart; caudal sections are 400 µm apart. Scale bar is 50 µm wide.

For every animal, four to six hippocampal sections separated by 1600 μm intervals were analysed using stereological methods. The person performing the quantification was blind to the treatment group to which the animals belonged. Image analysis was performed with Stereo Investigator software (MBF Bioscience, USA), connected to a LEICA DM LB microscope, equipped with a digital video camera (Optronics Microfire Digital Camera, U.S.A.) and ProScan II motorised stage system (Prior Scientific, U.S.A.). Hippocampal outlines were performed at 2.5x magnification (0.07 numerical aperture) and cell counting performed at 40x magnification (0.65 numerical aperture). HF borders were outlined on every analysed section according to the chick stereotaxic atlas^56^. Because of the complex structure of the avian HF, we divided the whole structure in two major components (Fig. 1): (i) the rostral HF (interaural 5.68 to 0.50) and (ii) the caudal HF (interaural 0.50 to −0.50).

Stereological parameters were set as follows: optical fractionator grid of 130 × 130 µm for the rostral HF and 260 × 260 µm for the caudal HF, counting frame of 70 × 70 µm for both regions and mounted thickness of 25 µm (accounting for 50% shrinkage). Different types of DCX^+^ cells have been previously described in avian brain literature^57^, and for our analysis we used a similar approach by defining two classes of cells according to neuronal morphology: (I) multipolar and (II) bipolar (fusiform). We also counted beaded axons (see Fig. 4 for examples of all categories). Multipolar cells were defined as medium-large sized cells, with a round or polygonal/angular cell body shape and process branching from it in three or more directions. Bipolar/fusiform cells were defined as medium-small sized cells with elliptical or oval cell body shape and process branching from it in two or fewer directions. Beaded axons were defined as small-sized points with process branching in two directions, and “necklace” shape strings of beads were counted as a single axon. All cells of these types lying inside the optical fractionator frame or bisected by its green lines were counted, according to the Optical Fractionator method.

The density of the different cell types was calculated by dividing the total number of cells of that type counted by the total volume of tissue sampled (number of counting grids times the volume of one counting grid (50 µm × 70 µm × 70 µm).

### Statistical analysis

Because staining intensity and background varied among staining batches, DCX expression was normalised by calculating the Standard Scores for each staining batch for densities of each cell class (Multipolar, Bipolar and Beaded Axons):

$${{\rm{Z}}}_{{\rm{i}}}=({{\rm{D}}}_{{\rm{i}}}-{\rm{M}})/{\rm{SD}}$$

In this, Di is the cell density of a particular cell type in either rostral or caudal HF, M is the Mean and SD is the Standard Deviation of all rostral and caudal HF densities for this cell type for a given staining batch. All analyses were conducted in IBM SPSS v24 (RRID:SCR_002865) and descriptive statistics are expressed as mean ± SEM. To control for plate-to-plate variation in the CORT ELISAs, we took an analogous approach to the AHN analysis, and normalized each titre to the mean and SD of the plate in which it was measured, using the same formula as above.

CORT and immunohistochemistry data were treated as linear and analysed without transformation using the Generalised Estimating Equations (GEE) model, with P values based on the Wald’s χ^2^ test statistics and parameters estimated by a maximum likelihood approach. Effects of time and UCMS condition on body mass were analysed via a repeated measures ANOVA, whilst univariate ANOVAs with final body mass as a covariate were used to compare endpoint organ masses between groups. Repeated measures ANOVAs over three time-points were conducted for H/L ratios, whilst independent sample t-tests were employed for qPCR data. TI times were analysed according to a Cox-regression survival analysis, with cases where immobility was sustained for ≥10 minutes included with a maximum duration of 600 seconds and non-event status in the analysis. Lastly, the number of attempts required to induce immobility at each time-point was explored using a GEE with a Poisson distribution. The least significant differences (LSD) post-hoc correction for multiple testing was utilised where appropriate, and P < 0.05 was considered statistically significant.

## Results

### Body and organ mass

Total body and whole organ masses of birds at tissue collection are displayed in Table 4. Body mass increased between arrival (age 15 weeks) and the end of the protocol (age 26 weeks) for all birds (*F*_4,34_ = 567.57, *p* < 0.001). There was no effect of UCMS on body mass (*F*_1,37_ = 0.010, *p* = 0.922), nor an effect limited to particular time-points (stress*time *F*_4,34_ = 0.337, *p* = 0.851). The mass of the spleen was significantly lower in birds exposed to UCMS than their control counterparts (*F*_1,36_ = 4.28, *p* = 0.046), whilst no group differences were observed in liver, pituitary or adrenal mass.

**Table 4:** Body and organ mass at the end of the experimental protocol for birds in the UCMS and control conditions.

Tissue	Control	UCMS	P^a^
Body mass (g)	1831 ± 130	1842 ± 121	0.922
Spleen (g)	2.3 ± 0.5	2.0 ± 0.5	0.046
Liver (g)	43.0 ± 5.5	41.7 ± 5.7	0.278
Pituitary (mg)	9.5 ± 2.5	9.5 ± 1.5	0.580
Adrenal (mg)	108.7 ± 29.2	108.8 ± 23.4	0.971

^a^Significance of UCMS effect, from a repeated measures ANOVA for body mass and univariate ANOVAs for organ masses, including body mass as a covariate (n = 39, mean ± SD).

### Tonic immobility

**Figure 2:**
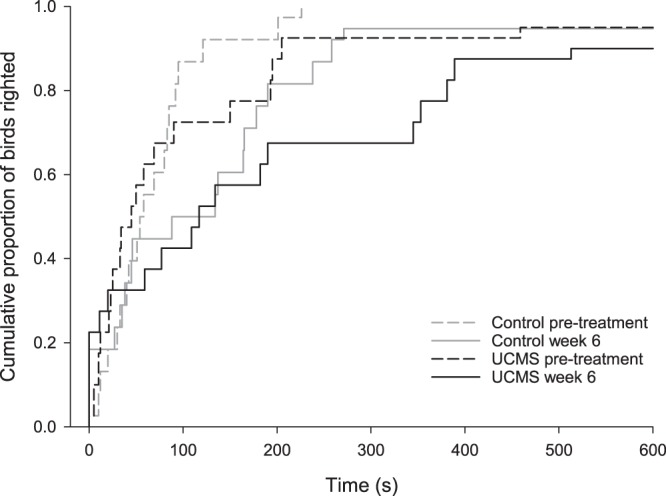
Survival distribution of time (s) until righting after induction of tonic immobility for birds in the control versus UCMS treatment groups at baseline & week 6 of UCMS.

A Cox regression was conducted with stress treatment group and time-point as covariates, in order to determine whether the survival distribution of time until righting differed for birds allocated to the UCMS and control conditions pre-treatment and during week 6 of UCMS treatment (Fig. 2). There was no main effect of allocated group (χ^2^(1) = 1.68, *p* = 0.195), but both treatment groups took longer to right themselves at the second time-point, compared to the first (χ^2^(1) = 4.47, *p* = 0.034). Treatment group did not interact with time-point (χ^2^(1) = 0.004, *p* = 0.951), indicating that 6 weeks of UCMS treatment did not increase time spent immobile more in comparison to the control group.

The number of attempts required to induce TI did not predict TI times (χ^2^(1) = 0.568, *p* = 0.451), and did not interact with time-point as a covariate (χ^2^(1) = 0.511, *p* = 0.475).

Significantly more attempts were required to induce TI during the experimental phase than pre-treatment for all hens (χ^2^(1) = 25.16, p < 0.001). There was no significant effect of stress treatment (χ^2^(1) = 0.735, p = 0.391), and stress treatment did not interact with time-point (χ^2^(1) = 1.38, p = 0.241). TI time did not significantly co-vary with number of attempts to induce the state (χ^2^(1) = 2.92, p = 0.087).

### Activity of the HPA-axis

**Figure 3:**
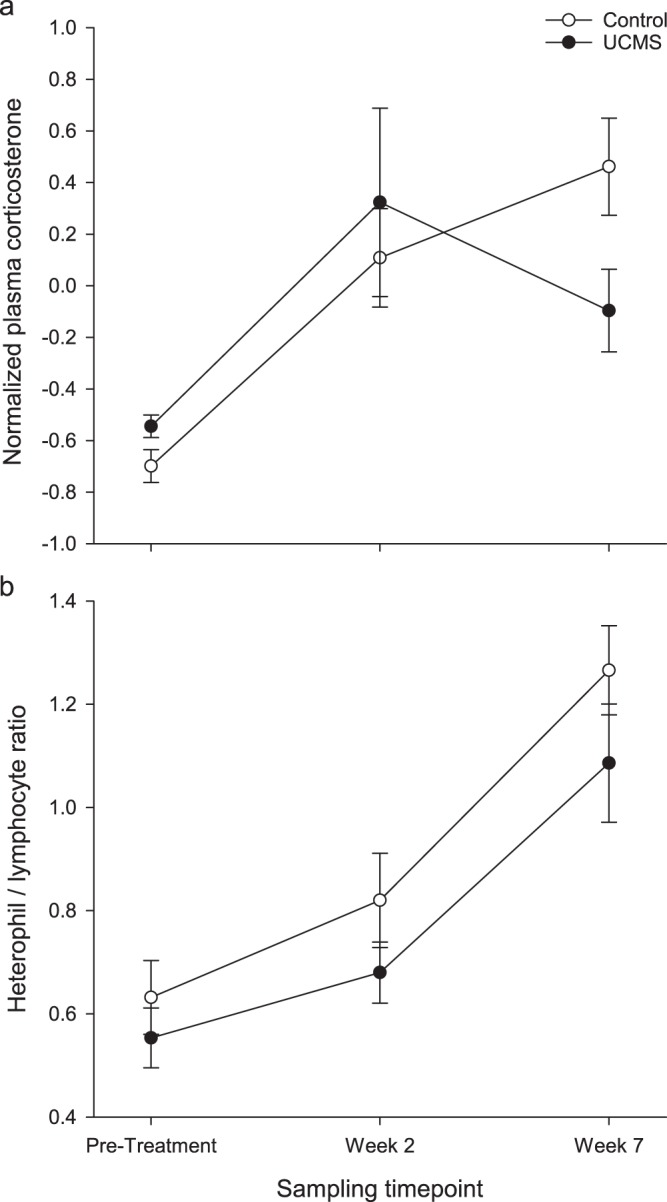
(**a**) Normalised plasma CORT titres and (**b**) ratio of heterophil to lymphocyte white blood cells, in samples taken from UCMS and control birds prior to treatment and during weeks two and seven of UCMS. Error bars = ± SEM.

For all birds, there was a significant main effect of time on plasma CORT titres (χ^2^(2) = 54.998, p < 0.001), which were lower in the pre-treatment than in week two and week seven of the experimental phase. There was no significant main effect of UCMS treatment on plasma CORT titres (χ^2^(1) = 0.186, p = 0.667), but there was an effect of stress dependent on time-point (stress*time χ^2^(2) = 7.107, p = 0.029, Fig. 3a), with the control group having higher baseline CORT titres than the stressed group in week 7, but no difference in week 2.

When expression of stress-related genes was measured in the adrenal gland, no significant effect of UCMS treatment was present for *MC2R* (t_35_ = 1.48, p = 0.149), *STAR* (t_35_ = 0.062, p = 0.951), *HSD3B2* (t_36_ = −0.528, p = 0.601) or *POMC*, (t_36_ = 0.850, p = 0.401).

Expression of *POMC* was further quantified in the pituitary gland, and was not influenced by UCMS in this tissue (t_36_ = 0.964, p = 0.341). Stress also did not modulate pituitary expression of *CRHR1* (t_34_ = 0.242, p = 0.810) or *VT4R* (t_36_ = 1.46, p = 0.154).

### Heterophil to lymphocyte ratio

The ratio of heterophil to lymphocyte white blood cells in plasma samples increased significantly over time for all birds, *F*_2,36_ = 25.03, p < 0.001 (Fig. 3b). H/L ratio was not significantly affected by UCMS treatment overall (*F*_1,37_ = 2.42, p = 0.128), nor in a time-point dependent manner (stress*time *F*_2,36_ = 1.62, p = 0.851).

### Morphological quantification of doublecortin

**Figure 4:**
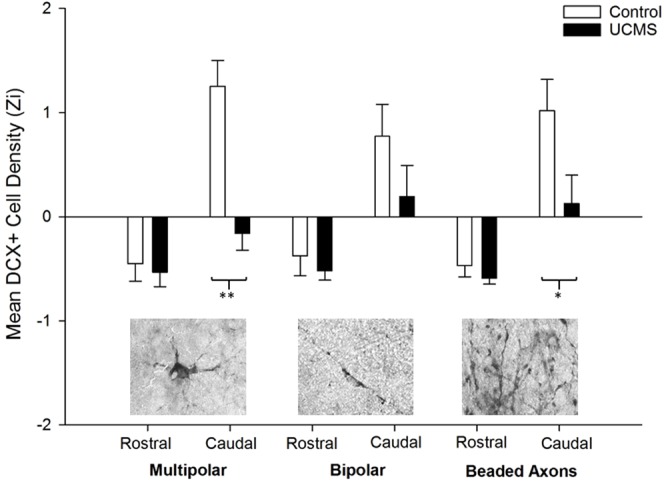
Mean density (Z-scored) of doublecortin-positive multipolar, bipolar and tiny cells in the rostral and caudal hippocampal sub-regions, following exposure to UCMS versus control conditions. Error bars = ± SEM. Asterisks indicate significant differences between conditions within a region (*0.05 < P > 0.01; **P < 0.01).

The density of DCX^+^ multipolar neurons was significantly lower in the HF of hens exposed to UCMS than control hens (χ^2^(1) = 12.97, *p* < 0.001). Whilst more multipolar neurons were present in the caudal hippocampal sub-region than the rostral area (χ^2^(1) = 55.85, *p* = < 0.001), a stress by sub-region interaction was also observed (χ^2^(1) = 22.99, *p* < 0.001, Fig. 4). Pairwise comparisons indicated that at the caudal pole, DCX^+^ multipolar cell density was significantly reduced following exposure to UCMS (M = −0.160, SEM = 0.154) compared to control conditions (M = 1.25, SEM = 0.238, p < 0.001). In contrast, DCX^+^ multipolar cell density in the rostral HF did not differ between UCMS and control groups (p = 0.709). Whilst control hens had a higher density of stained multipolar cells in the caudal than in the rostral HF (p < 0.001), there was no subregional difference for the UCMS group (p = 0.056).

Whilst the density of DCX^+^ bipolar neurons was also higher in the caudal hippocampus than in the rostral region (χ^2^(1) = 14.16, *p* < 0.001), it did not differ significantly according to UCMS treatment (χ^2^(1) = 3.04, *p* = 0.081). The interaction between stress treatment and sub-region was not significant (χ^2^(1) = 0.767, *p* = 0.381).

Density of DCX^+^ beaded axons was significantly lower in hens exposed to UCMS (M = −0.232, SEM = 0.126) than control hens (M = 0.274, SEM = 0.162; χ^2^(1) = 6.06, *p* = 0.014). Beaded axon density was higher in the caudal hippocampus (χ^2^(1) = 31.22, *p* < 0.001) and an interaction existed between sub-region and UCMS condition, (χ^2^(1) = 3.86, *p* = 0.049). At the caudal pole, hens exposed to UCMS had a significantly lower density of beaded axons than control hens (p = 0.021), whereas density in the rostral region did not differ between conditions (p = 0.314). Both control hens and hens exposed to UCMS had a higher density of beaded axons in the caudal hippocampus than the rostral (control: p < 0.001; UCMS: p = 0.010, Fig. 4).

## Discussion

### Hippocampal homologies

Following 8 weeks of unpredictable chronic mild stress (UCMS), a reduction in the density of DCX^+^ multipolar neurons occurred selectively at the caudal pole of the chicken HF. Though non-significant, a similar pattern in mean densities was observed for bipolar cells. This pattern confirms our anatomy-driven hypothesis that the caudal pole of the avian HF is homologous to the ventral rodent hippocampus, where selective reductions in AHN following chronic stress have also been reported^17–21,37^. Together with the previous observation that electrical stimulation of the caudal pole of the avian HF suppresses HPA-axis activity more powerfully than at rostral sites^35^, a clear role is suggested for the caudal avian HF in affective modulation. Whether adult-born cells in the chicken HF have a similar role in inhibiting stress-responsive mature neurons in the caudal subregion, as occurs in the ventral dentate gyrus of mice^28^, remains to be explored. In addition, homologous sensitivity of AHN in avian and rodent brains to chronic stress suggests that the AHN in modern mammals derives from a process established in their common ancestor with birds over 300 million years ago^31^.

We found that, in general, there was a higher density of DCX^+^ neurons in the caudal than in the rostral HF. This finding is similar to findings in zebra finches^58^ and in two species of North American blackbirds^59^. However, wild-caught chickadees tend to generate more new neurons in the rostral HF than the caudal HF, although the ones in caudal HF have a longer turn-over time. Interestingly, the rostro-caudal gradient in chickadees is eliminated once they are kept in captivity for 6 weeks^40^. In our own lab, we found more dividing cells (BrdU^+^ cells in the ventricular zone) in the caudal than in the rostral HF in broiler breeder chickens, but no overall differences in new neuron density (BrdU^+^Hu^+^) between the two poles of the HF (Robertson *et al*. 2017). In mice, we found similar numbers of new neurons in the dorsal and ventral dentate gyrus of mice in standard housing. However, when animals were kept in enriched environments for 8 days, the number of new DCX^+^ neurons in the dorsal hippocampus went up, and those in the ventral hippocampus was reduced^55^. In the hippocampus of singly-housed adult rats, density of both four-week-old BrdU^+^ cells and immature PSA-NCAM^+^ cells was also higher in the dorsal than the ventral dentate gyrus^60^. It is clear, therefore, that the pattern of neurogenesis across the dorsoventral axis in rodents, and the rostrocaudal axis in birds can depend on a variety of factors, from the animals’ experiences to differences between species and even possibly different methods of labelling new neurons.

We quantified two cell types: bipolar (fusiform) neurons and multipolar neurons. Like Boseret *et al*.^57^, we assume that the fusiform neurons are younger and still migrating, while the multipolar neurons are more mature and settling. Based on Golgi analysis of the chick HF^61^, the latter group included a high proportion of multipolar projection neurons, but may have also incorporated pyramidal and multipolar local circuit neurons. In the mouse dentate gyrus, newly generated granule cells are functionally integrated into the neural circuitry by 4 weeks^62^, and as DCX is expressed for around 4 weeks in birds^47^, some of the multipolar cells we stained were likely functional. The beaded axons we counted very likely belong to multipolar projection neurons (see^61^), which may explain why their densities co-varied within subregions and UCMS treatments.

The density of DCX^+^ multipolar neurons was selectively suppressed at the caudal HF pole following UCMS, and whilst this pattern existed for the bipolar neurons as well, it was not significant in the still-migrating cells. UCMS therefore resulted in the survival of fewer developing neural cells to a later stage of maturity. Previous studies have similarly demonstrated a stronger influence of chronic stress on later stages of neurogenesis, with both UCMS protocols in adult rats^63,64^ and chronic restraint in mice^65^ acting to decrease the survival of new-born hippocampal cells without attenuating proliferation. However, other studies have reported chronic stress-driven suppression of both proliferation and survival, for example following chronic social defeat in rats^66^ and restraint in mice^67^. Inconsistent results are likely dependent upon characteristics of the chronic stress paradigms, such as their predictability, and differing markers of AHN utilised (e.g. BrdU, Ki67, PCNA, DCX), given that these interact with experimental timings to determine the particular cellular population quantified^64^.

### Implications for animal welfare monitoring

In addition to the clear effect on AHN, exposure to UCMS led to a decrease in baseline plasma CORT titres relative to control birds and in relative spleen mass by the final time-point. Previous research has demonstrated similarly reduced baseline CORT levels in avian species undergoing chronic stress, including wild-caught chukar (*Alectoris chukar*) monitored after transfer to captivity^68^ and captive European starlings (*Sturnus vulgaris*) exposed to a randomised, unpredictable sequence of stressors^69^. Though chronic stress is typically expected to increase HPA activity and thus CORT release, the latter authors infer a mechanism of physiological adaptation to prolonged challenge, which may be triggered by unpredictable protocols designed to prevent habituation^69^. The spleen, as well as other immune function organs, such as the thymus and the Bursa of Fabricius, is known to shrink with chronic exposure to increased CORT levels, be it directly manipulated or in response to artificial increase in ACTH^70,71^. If we assume, like other authors before us, that the reduced baseline of CORT is a response to repeated stimulation of the HPA axis, then the reduction in spleen mass is consistent with this chronic exposure to (repeatedly elevated) CORT titres. This validates the efficacy of our UCMS protocol.

Arguably, some aspects of the UCMS protocol, such as social mixing and greater overall human contact, could be considered enriching, but it is unlikely that components such as exposure to water and fans, removal of perches and food deprivation would induce a positive response. Furthermore, whilst CORT plays a causal role in regulating neurogenesis, it is the valence of experience that determines whether AHN is increased or decreased^72^, and the observed reduction in new neurons does not support the notion that the birds found the conditions enriching, given that enrichment robustly upregulates AHN^16^. We do not know which aspects of the UCMS protocol had a strong effect on the birds’ affective states compared to the others. One of our stressors was occasional 12-hour food deprivation. Since food restriction is associated with reduced AHN in young female broilers^45^, it is theoretically possible that this factor was the main contributor to our stress effect. However, neither growth nor egg production in the UCMS group suffered compared to control birds, suggesting the sporadic food deprivation did not constitute a major nutritional stressor comparable to restricted dietary regimes in broiler breeders^73^. In addition, the severe and chronic food restriction experienced by broiler breeders increased baseline CORT levels, while our treatment decreased them. Therefore, we find it unlikely that food deprivation is solely responsible for the observed effect, and though it is not clear which of the multiple stressors employed contribute most to reduced AHN, their cumulative impact is clear.

Compared to pre-treatment, plasma CORT titres were higher at weeks two and seven of the experiment for both UCMS and control birds. As birds had already been introduced to their new pens prior to pre-treatment blood sampling, this increase is unlikely to reflect a collective response to social stress. However, egg production per pen reached its peak during week two before plateauing, meaning this was the approximate age that hens reached sexual maturity (~20 weeks). As CORT production has previously been reported to increase around the onset of lay^74^, this likely explains the increase in plasma levels that occurred over time in both groups. H/L ratios were not responsive to stress treatment, and they also increased over time for both UCMS and control groups to be highest in the penultimate week of the experimental phase, when birds were 25 weeks old. Previous authors have reported a similar age effect on H/L ratio in female chickens, with this increasing incrementally between sexual maturity at age 20 weeks (corresponding here to UCMS week 2) and age 28 weeks^75^.

Durations of TI are believed to reflect anxiety state^51^. However, they were not differentially influenced by UCMS treatment. They were, however, longer at week six of the protocol than pre-treatment for all birds and more attempts were required to induce TI at the later time-point, which may reflect some degree of habituation to the test procedure by the third session, as has been observed previously in adult birds^76^. Therefore, whilst baseline plasma CORT and AHN proved responsive to UCMS in adult laying hens, H/L ratios and TI durations appear less sensitive. A lack of change in these latter traditional measures may also relate to the relative stress resilience of modern brown strains, which are more docile^77^ and less fearful of humans^78^ than white strains. Indeed, Hyline Brown hens exhibit shorter durations of tonic immobility and a reduced plasma CORT response to acute handling stress compared to the White Leghorn^79^.

We also measured basal expression of genes central to the physiological stress response within the adrenal-axis and found no influence of UCMS. These included those involved in glucocorticoid synthesis (*STAR*, *HSD3B2*), one encoding the signalling molecule which triggers this process (*POMC*, encoding adrenocorticotropic hormone, ACTH), and receptors for ACTH and its hypothalamic secretagogues (*CRHR1*, *MC2R* and *VT4R*). Whilst acute restraint stress in laying hens is known to upregulate adrenal expression of *POMC* and *STAR*^49^, the lack of change here may be attributable to the delay between acute stressors and tissue sampling, which suggests that altered expression of this mRNA under acute stress is transient and not sustained in a protocol that is chronic but intermittent. Previous work has also found that pituitary expression of *VT4R* and *CRHR1* is differentially affected by chronically recurring as compared to acute immobilisation stress^50^, when measured immediately following the final stressor. That a similar response to our UCMS protocol did not occur suggests either that gene expression during the acute stress response, but not between stressors, is altered in the chicken adrenal-axis by chronic stress, or that expression of these genes is differentially impacted by an unpredictable sequence of different stressors than by repeated exposure to the same stressor. Collecting tissues from some animals directly following an acute stressor might clarify this potential discrepancy in future UCMS protocols. This does not take away, however, from the clear effects of chronic, cumulative stress on baseline CORT and on AHN.

Overall, our findings suggest that post-mortem quantification of AHN in poultry, particularly in the caudal HF, provides a measure of cumulative chronic stress, and therefore welfare state, that is more sensitive to a sequence of negative experiences than certain commonly-employed behavioural and physiological indices, as well as adrenal-axis gene expression. This may apply particularly to modern commercial strains, such as Hyline brown, which display lesser responses for traditional measures of stress^79^. Such a sensitive measure could be applied to key welfare comparisons within the poultry industry, for example assessing the relative cumulative experience of focal birds kept in different commercial housing systems, or subject to alternative husbandry practices, when they are killed at the end of the production cycle. Whilst it would not be possible to distinguish which aspects of one complex set of housing conditions were responsible for greater stress, the cumulative nature of the overall experience would be apparent. More experimentally, by changing single factors predicted to have a major effect on welfare, the efficacy of such interventions could be assessed using AHN. This method could thus be used to optimize laying hen housing systems for the highest possible welfare, a highly desirable outcome in many Western societies.

## Conclusions

In conclusion, the caudal pole of the laying hen HF is preferentially sensitive to the experience of unpredictable chronic stress, and the number of newly-generated neurons surviving, potentially to the point of functional integration, in this region is reduced in line with this negative welfare experience. This finding supports a comparable modulation of adult neurogenesis in the avian HF by stress to that observed in rodents. Furthermore, a homologous functional gradient may exist along the rostrocaudal axis of the avian HF, wherein the caudal pole is homologous to the ventral rodent hippocampus and involved more heavily in stress modulation. The impact of our UCMS treatment was also supported by altered baseline plasma CORT titres and the reduced spleen mass in stressed hens by the final time-point. In contrast, gene expression in the HPA axis was not modified following chronic unpredictable stress and H/L ratios and TI times also did not reflect this experience. Thus, measuring neurogenesis in the caudal HF post-mortem may provide a sensitive measure of cumulative welfare state in poultry, which could allow comparison of stress engendered by different commercial housing systems. Whether AHN in the poultry HF is also sensitive to positive experiences, as it is in rodents, remains to be seen.

## Data Availability

The datasets generated during and/or analysed during the current study are available from the corresponding author on reasonable request.
